# NLRP3 inflammasome activation from Kupffer cells is involved in liver fibrosis of *Schistosoma japonicum-*infected mice *via* NF-κB

**DOI:** 10.1186/s13071-018-3223-8

**Published:** 2019-01-11

**Authors:** Wen-Juan Zhang, Zheng-Ming Fang, Wen-Qi Liu

**Affiliations:** Department of Parasitology, School of Basic Medicine, Tongji Medical College, Huazhong University of Science and Technology, Wuhan, 430070 Hubei People’s Republic of China

**Keywords:** NLRP3 inflammasome, Liver fibrosis, *Schistosoma japonicum*, Kupffer cells

## Abstract

**Background:**

NOD-like receptor protein 3 (NLRP3) inflammasome was reported as expressed in schistosomiasis-induced liver fibrosis (SSLF). We used an NLRP3 inflammasome inhibitor, MCC950, to investigate whether it inhibited liver fibrosis, and explored the preliminary molecular mechanism.

**Methods:**

BALB/c mice were infected with 15 cercariae through the abdominal skin. They received intraperitoneal injections of MCC950 on the day of infection and at day 22 post-infection. We examined their SSLF phenotype and the effect on liver fibrosis, primary Kupffer cells (KCs), and HSCs. Human hepatic stellate cell lines (human LX-2 cells) were treated with soluble egg antigen (SEA) released from the eggs. We then determined the expression of NLRP3 inflammasome and liver fibrosis-associated markers, liver granuloma and ALT/AST.

**Results:**

NLRP3 inflammasome expression in the liver was significantly increased, and eosinophilic granuloma and collagen deposition were found around the eggs in mice infected for 56 days. Additionally, IL-1β, ALT/AST in plasma, and NF-κB in liver tissue and in KCs were all greatly significantly increased. The above-mentioned indicators were largely reduced in mice treated with MCC950 on the day of infection. *In vitro*, lipopolysaccharide (LPS)/SEA could induce LX-2 cells to express NLRP3 and fibrosis markers, and the SEA-treated group was reversed by MCC950. Furthermore, NLRP3 inflammasome and liver fibrosis-associated markers were both increased in the primary KCs and HSCs isolated from infected mice. However, this effect was not observed in the same cells from the mice treated with MCC950 on the day of infection. Contrary to the aforementioned results, MCC950 treatment at day 22 post-infection aggravated this process. Surprisingly, NLRP3 inflammasome was involved in liver fibrosis mostly from KCs.

**Conclusions:**

MCC950 acts dually on SSLF pathology and fibrosis in infected mice. Although MCC950 treatment improved SSLF on the day of infection, it exacerbated the pathological effects at day 22 post-infection. These dual effects were mediated *via* NF-κB. Moreover, NLRP3 inflammasome mainly came from KCs. Our results suggest that blocking NLRP3 on the day of infection may prove to be a promising direction in preventing SSLF.

## Background

Schistosomiasis is a helminthic infectious disease and is prevalent in Asia, Africa and Latin America, where the susceptible population has reached 200 million people and spread to 74 countries and regions [1, 2]. Liver fibrosis is the main pathological feature of *S. japonicum* infection [1, 3]. The immune reaction and inflammatory response is caused by soluble egg antigen (SEA) released from schistosome eggs. Hepatic stellate cells (HSCs) activation is vital for liver fibrosis in schistosomiasis [3–6]. Kupffer cells (KCs), as immune cells, can quickly sense hepatic injury and produce inflammatory cytokines or chemokines such as interleukin 1β (IL-1β), tumor necrosis factor-α (TNF-α) or transforming growth factor-β (TGF-β) [7–10]. These factors could contribute to HSCs proliferation and activation, and then the extracellular matrix (ECM) synthesis, and could result in liver fibrosis [11]. The multiple cytokines secreted by HSCs could recruit KCs and subsequently promote KCs to release more inflammatory mediators in turn, leading to the cell-cell interactions with one another [12, 13]. As an injury stimulating factor, SEA could initiate HSCs activation and transformation into myofibroblasts, which could ultimately develop into collagen and other ECM component deposition in the liver. This results in granulomas and hepatic fibrosis surrounding the eggs [14, 15].

NLRP3 inflammasome is a multi-protein complex and a natural component of the immune system. It usually participates in the pathogenesis of inflammatory responses with pro-cysteinyl aspartate specific proteinase (pro-caspase I) and pro-IL-1β, involved in human liver disease [16–18]. In particular, resident macrophages or KCs become activated and trigger an inflammatory response through common pathways of the NLRP3 inflammasome and IL-1β [19]. Growing evidence has linked NLRP3 inflammasome-driven inflammation to tissue damage and liver fibrosis in conditions such as drug-induced liver injury, alcoholic steatohepatitis (ASH) and angiotensin II (Ang-II) [20, 21]. In fact, recent studies have found that NLRP3 inflammasome is present in liver fibrosis induced by *S. japonicum* infection [17, 22]. NLRP3 inflammasome is composed of NLRP3 protein, apoptosis associated speck-like protein (ASC) and pro-caspase I [19, 23]. NLRP3 serves as a backbone protein of the complex and ASC acts as a linker protein linking NLRP3 with pro-caspase I. The NLRP3 inflammasome can be recognized by extracellular pathogen-associated molecular patterns (PAMPs) and intracellular danger-associated molecular patterns (DAMPs), and it activates downstream signaling pathways by pattern recognition receptors (PRRs) on the cell surface [24–26]. Recently, studies have shown that a western diet can evoke NLRP3 inflammasome activation in liver fibrosis [27]. NLRP3 inflammasome activation traditionally requires a dual signal. The first signal is mostly from TLR activation for inflammasome expression and the second signal comes from the inflammasome ligand for inflammasome activation [28]. After the NLRP3 inflammasome forms, pro-caspase I could then be activated to caspase I. This, in turn, leads to pro-IL-lβ and pro-IL-18 to be cleaved and to enter their activated forms [23, 24]. These cytokines could activate KCs, which then mediate HSCs activation and promote liver fibrosis [29]. Nuclear factor-κB (NF-κB), a transcription factor, regulates the formation of NLRP3 inflammasome [28, 30] and takes part in the progression of liver fibrosis [31]. NF-κB could affect NLRP3 inflammasome in two ways. One way is through TLR induced NLRP3 inflammasome activation *via* NF-κB [32] and the other is through NF-κB elimination of damaged mitochondria *via* autophagy receptor p62, which eventually inhibits activation of NLRP3 inflammasome [30]. In recent years, NF-κB has been shown to play a negative regulatory role in NLRP3 inflammasome *via* ubiquitin-modifying enzyme A20 [33, 34]. Together, these findings indicate that NLRP3 inflammasome and NF-κB are both implicated in liver fibrosis.

Currently, a large number of studies focus on NLRP3 inflammasome expression and its activation mechanisms [35, 36] but few have examined how NLRP3 inflammasome regulates liver fibrosis [7, 22]. Here, we aim to provide more data to deeply understand the effects of NLRP3 on the process of liver fibrosis in schistosomiasis. NLRP3 inflammasome is widely expressed in a variety of cells in the liver, including KCs, HSCs and hepatocytes [16]. KCs [37] and HSCs [4] play a key role in liver fibrosis and are both often used to explore liver development. However, the question of which cells are responsible for NLRP3 inflammasome-mediated liver fibrosis remains unanswered.

The NLRP3 inflammasome inhibitor MCC950 (also known as CP-456,773), has been reported to counteract the LPS-induced increase of IL-1β and IL-6 in serum from an autoimmune encephalomyelitis (EAE) mouse model [38]. As far as we know, liver fibrosis of *S. japonicum* is different from other liver fibrosis models because the host is affected by both the growth of the worm and the stimulation of the eggs. Therefore, MCC950 could block NLRP3 inflammasome at different time points, enabling us to observe hepatic granulomas and fibrosis caused by *S. japonicum* infection. The main purpose of this study was to use NLRP3 inflammasome inhibitor, MCC950, to block liver granuloma and fibrosis that resulted from *S. japonicum* in order to uncover the underlying molecular mechanism and potential treatments for schistosomiasis. To address these issues, we used mouse models to detect NLRP3 inflammasome and pro-fibrosis markers, as well as LX-2 cells, primary KCs and HSCs from mice.

## Methods

### Animals, *S. japonicum* infections and reagents

BALB/c mice (6–8 weeks-old, female, body weight 18–20 g) were purchased from Hubei Provincial Center for Disease Control and Prevention (Wuhan, China). All mice were bred in a specific pathogen-free (SPF) condition. *Oncomelania hupensis* snails were provided by Nanjing Institute of Schistosomiasis Prevention and Control (Nanjing, China). Mice were randomly divided into four groups, with six mice in each group. In the SJ group, after the mice were anesthetized, they were infected with 15 *S. japonicum* cercariae through the abdominal skin. In the M0 group, the mice were intraperitoneally (i.p.) injected with MCC950 (Sigma-Aldrich, St. Louis, USA, 10 mg/kg) starting from the day of infection, days 1 and 2, and then every 2 days until day 56 post-infection. In the M4 group, the mice were given i.p. injections of MCC950 (10 mg/kg) at day 22 post-infection, days 23 and 24, and every 2 days thereafter until day 56 post-infection. In the control group, the mice were injected with vehicle (0.9% NaCl) at the same time points.

*Schistosoma japonicum* SEA was prepared from purified eggs in the livers of *S. japonicum*-infected rabbits (1500 ± 100 cercariae/rabbit) and sterile filtered *via* a 0.22 μm filter (Millipore, Bedford, MA, USA), as previously described [39] with a minor modification. Briefly, eggs of *S. japonicum* were collected from infected rabbit livers. The rabbits were sacrificed, and liver tissues were separated. The rabbit livers were cut and ground into pieces. The eggs were collected through coarse screening, digesting, fine screening, cleaning and purifying. Eggs were transferred to a 0.9% NaCl solution, then homogenized for 20 min on ice, and received a freeze/thaw treatment at least three times. The SEA was centrifuged at 14,000× *g* for 20 min at 4 °C, and the supernatant was collected. A BCA protein assay kit was used to determine protein concentration (Beyotime, Shanghai, China). SEA (1 g/ml) was diluted with phosphate-buffered saline (PBS) for storage or diluted into working concentration with Dulbecco’s modified Eagle’s medium (DMEM) containing 2% fetal bovine serum (FBS) before use.

### Sample preparation, liver and spleen indexes, and worm and egg burden examination in the livers

Mice were weighed once per week until the end of the experiment. At the end point, all mice were sacrificed and blood, livers, spleens and mesenteric tissues were collected to perform pathological and molecular biological analysis. The livers and spleens were weighed and 1–2 liver fragments (0.5 × 0.5 × 0.5 cm) were soaked in 4% paraformaldehyde (PFA). The remaining fragments were placed into tubes, flash frozen in liquid nitrogen, and stored at -80 °C. The mesenteric tissues were placed in ice-cold normal saline solution containing sodium heparin (1%).

The liver and spleen indexes were calculated according to liver/spleen weight *versus* body weight. Adult worms located in the mesenteric veins were recorded. To estimate the egg burden, 0.2 g of each liver was digested with 20 ml KOH (10%) for 3 h at 37 °C, and the numbers of eggs were counted under a microscope. Total eggs per gram (epg) in the liver were calculated using the following formula: epg = the number of eggs calculated × 5.

### Analyses of liver fibrosis, liver function, pathology and immunohistochemical staining

The serum was obtained by centrifugation of mouse blood at 3500× *rpm* for 15 min at 4 °C to examine liver function and IL-1β. Alamine aminotransferase (ALT), aspartate aminotransferase (AST) and hepatic hydroxyproline (Hyp) content were tested using commercial kits (Nanjing Jiancheng Bioengineering Institute, Nanjing, China) according to the kit instructions.

The liver tissues were embedded in paraffin and sliced into 4 μm sections. Hematoxylin-eosin (HE) staining analyzed the area of granuloma. Masson staining analyzed the severity of collagen deposition. After HE and Masson stainings, 3 different fields under a microscope with 200× magnification were randomly selected from each sample for analysis. The area of the granulomas in HE-staining was quantified using the Mshot Image Analysis System (Micro-shot Technology, Guangzhou, China). In addition, the percentage of each slide area was positive for the blue in Masson staining, which was then analyzed using Image-Pro Plus 6.0 software (Media Cybernetics, Bethesda, MA, USA). The reagents in immunohistochemical staining are summarized in Table 1. Samples were both blocked with 3% hydrogen peroxide (H_2_O_2_) for endogenous peroxidase and 3% bovine serum albumin (BSA) for non-specific binding sites of the primary antibody. The samples were incubated with primary antibodies (Table 1), such as goat anti-NLRP3 (1:200), rabbit anti-Caspase-1 (1:200) and goat anti-IL-1β (1:100) overnight at 4 °C, and then washed 3 times in PBS. They were then incubated with rabbit anti-goat or goat anti-rabbit HRP-conjugated secondary antibodies (1:500; Earthox, CA, USA) (Table 1) at room temperature for 1 h, then washed 3 times in PBS. For the color reaction of HRP, streptavidin-peroxidase complex 3, 3-diaminobenzidine tetrahydroxychloride (DAB) was used as a substrate. Negative controls were not added to the primary antibody. A total of 3 fields per sample were examined. Positive immunostainings were quantified as integrated optical density (IOD) using the Image-Pro Plus6.0 software.

**Table 1:** Antibodies and reagents used for immunohistochemistry and immunofluorescence

Primary antibody	Antibody type	Dilution buffer (dilution)	Company	Secondary antibody	Dilution buffer (dilution)	Blocking buffer	Wash buffer (time × repetitions)
NLRP3	Goat *vs* mouse NLRP3 (polyclonal)	PBS (1:200)/5% BSA in PBS (1:200)	Abcam, Cambridge, UK	Rabbit *vs* goat HRP-labeled/donkey *vs* goat FITC- labeled	PBS (1:500)/5% BSA in PBS (1:50)	3% H_2_O_2_and 3% BSA	PBS (5 min × 3)
Caspase-1	Rabbit *vs* mouse Caspase-1 (polyclonal)	PBS (1:200)	Abcam, Cambridge, UK	Goat *vs* rabbit HRP-labeled	PBS (1:500)	3% H_2_O_2_and 3% BSA	PBS (5 min × 3)
IL-1β	Goat *vs* mouse IL-1β (polyclonal)	PBS (1:100)	R&D, Minneapolis, USA	Rabbit *vs* goat HRP-labeled	PBS (1:500)	3% H_2_O_2_and 3% BSA	PBS (5 min × 3)
NF-κB	Rabbit *vs* mouse NF-κB (polyclonal)	5% BSA in PBS (1:200)	Abcam, Cambridge, UK	Goat *vs* rabbit FITC-labeled	5% BSA in PBS (1:50)	3% H_2_O_2_and 3% BSA	PBS (5 min × 3)
F4/80	Rabbit *vs* mouse F4/80 (polyclonal)/	5% BSA in PBS (1:100)/	Bioss, Beijing, China	Goat *vs* rabbit Cy3-labeled/	5% BSA in PBS (1:50)	3% H_2_O_2_and 3% BSA	PBS (5 min × 3)
	Mouse *vs* mouse F4/80 (monoclonal)	5% BSA in PBS (1:50)	Santa, California, USA	Goat *vs* mouse Cy3-labeled			
α-SMA	Mouse *vs* mouse α-SMA (polyclonal)	5% BSA in PBS (1:200)	Abcam, Cambridge, UK	Goat *vs* mouse Cy3-labeled	5% BSA in PBS (1:50)	3% H_2_O_2_and 3% BSA	PBS (5 min × 3)

*Abbreviations*: *NLRP3* nod-like receptor protein-3, *Caspase 1* cysteinyl aspartate specific proteinase, *IL-1β* interleukin, *NF-κB* nuclear factor-κB, *F4/80* mouse epidermal growth factor-like module-containing mucin-like hormone receptor-like 1, *α-SMA* α-smooth muscle actin

### Separation of mouse primary KCs and HSCs

The main solutions were prepared according to the following formulas: Solution I: 0.05% Collagenase IV (Sigma-Aldrich); Solution II: 0.2% Collagenase IV + 0.2% Pronase E (Yuanye, Shanghai, China).

In selected experiments, we isolated and cultured KCs and HSCs from mice. The primary KCs and HSCs were separated by a collagenase perfusion method and a discontinuous density gradient centrifugation technique [40, 41]. In brief, the livers were perfused with PBS, then with two kinds of collagenase through the portal veins. The remaining liver tissues were cut into small pieces and suspended in PBS. After filtration through a 200-gauge mesh, the supernatant was separated from the collected cell suspension by centrifugation at 50× *g* for 3 min, then centrifuged at 500× *g* for 5 min to pellet the KCs and HSCs. The pellets were resuspended in PBS and then added to a 30/70% Percoll non-continuous density gradient separation liquid upper layer (Solarbio, Beijing, China) and centrifuged at 900**×** *g* for 15 min. The KCs and HSCs were collected from the interface of 30% and 70% Percoll, PBS and 30% Percoll, respectively. The two kinds of cells were both resuspended in mouse liver Kupffer cell and hepatic stellate cell medium (DMEM containing 10% heat-inactivated FBS, 1% glutamine and 1% penicillin-streptomycin) (Procell, Wuhan, China), respectively. They were centrifuged at 400× *g* for 5 min to remove the remaining Percoll solution in the cells.

### Cell culture and *in vitro* treatment

The collected mouse primary cells were resuspended in mouse liver Kupffer cell and hepatic stellate cell complete medium, then plated in six-well plates coated with Poly-L-Lysine (Procell, Wuhan, China) and cultured in a humidified incubator at 37 °C with 5% CO_2_. The KCs were cultured 2–3 h for further purification. The medium was changed after 3 h and the following day. Cells were then obtained on the third day. KCs were examined for their staining with mouse epidermal growth factor-like module-containing mucin-like hormone receptor-like 1 (F4/80). HSCs were cultured 24 h for further purification. The medium was changed after 24 h and on the third day. Cells were then collected on the fourth day. HSCs were identified by their staining with α-smooth muscle actin (α-SMA). Immunofluorescence was used for staining cells. Both the purity and viability of the cells were greater than 90% (Trypan Blue exclusion).

LX-2 cells (human HSCs line) were acquired from the Cell Collection Center of Wuhan University (China). The cells were derived from normal human HSCs and had the property of immortalized proliferation. The cells were cultured in DMEM (Gibco-Invitrogen, Carlsbad, CA, USA) containing 10% heat-inactivated FBS (Gibco, Carlsbad, CA, USA) and 1% penicillin-streptomycin (Life Technologies, Leuven, Belgium) at 37 °C in an atmosphere with 5% CO_2_. The cultures were passaged with 0.05% trypsin-EDTA (Gibco) every one or two days, and studies were performed during the fourth to seventh passages *in vitro*. For all experiments, LX-2 cells (5 × 10^5^ cells/well) were plated in six-well plates and cultured for 24 h. The cells were serum-starved for 12 h before treatment.

LX-2 cells were treated with SEA (10 μg/ml) at the appropriate time. In certain experiments, the cells were pre-treated for 4 h with NLRP3 inflammasome inhibitor, MCC950 (1 μM), to inhibit the corresponding target. Lipopolysaccharide (LPS) (100 ng/ml, Sigma-Aldrich) was used as a positive control. PBS was added as a vehicle control. The optimal expression of NLRP3 protein in our experiment followed SEA stimulation for 24 h and LPS stimulation for 2 h. Subsequent experiments were similar.

### Immunofluorescence staining

Immunofluorescence was performed on 2 μm sections of paraffin-embedded liver tissues, LX-2 cells, primary mouse KCs and HSCs seeded on six-well chamber slides. They were then fixed in 4% PFA, permeabilized with 0.15% Triton X-100, and blocked with 3% H_2_O_2_ and 3% BSA. Primary antibodies were used for immunofluorescence double staining (Table 1): goat anti-NLRP3 (1:200) with rabbit anti-F4/80 (1:100) or with mouse anti-α-SMA/ACTA2 antibody (1:200), and rabbit anti-NF-κB (1:200) and mouse anti-F4/80 (1:50) were incubated overnight at 4 °C. Then, the samples were washed 3 times in PBS. Fluorescein isothiocyanate (FITC) (green, 1:50, ProteinTech Group, Chicago, USA) labeled donkey anti-goat and goat anti-rabbit, or cyanine 3.18 (Cy3) (red, 1:50, Aspen, Wuhan, China) labeled goat anti-rabbit and goat anti-mouse secondary antibodies (Table 1) were used for 50 min at room temperature. The samples were washed 3 times in PBS again. Nuclei were stained with DAPI. Fluorescent images were taken using an inverted fluorescence microscope (MF53 Mercury, Guangzhou, China). The co-localization area percentage of NLRP3 *vs* F4/80 or α-SMA, and NF-κB *vs* F4/80 in specific target cells of liver tissues was analyzed, along with integrated optical density (IOD) for target protein in cells by Image-Pro Plus 6.0 software.

### Real-time PCR

Total RNA was extracted from cells and liver tissues by Trizol (Invitrogen, Carlsbad, CA, USA) and quantified by a spectrophotometer. In total, 2 μg RNA was reverse-transcribed to cDNA *via* the PrimeScript RT reagent kit (Toyob, Osaka, Japan), and real-time PCR (RT-PCR) was carried out using SYBR green master mix (Toyob) on a MyiQTM2 (Bio-Rad, Hercules, CA, USA). PCR primers were designed by a primer blasting tool on the NCBI website; primer sequences are summarized in Table 2. The glyceraldehyde-3-phosphate dehydrogenase (GAPDH) housekeeping gene was used as a reference control. The relative changes in gene expression were calculated by the 2^−ΔΔCT^ method.

**Table 2:** Sequence of primers used for quantitative RT-PCR

Gene	Species	Primer sequences (5'-3')	Annealing T (°C)	Length (bp)	GenBank ID
NLRP3	Mouse	F: GACCAGCCAGAGTGGAATGAC	60	237	NM_145827.3
		R: CTGCGTGTAGCGACTGTTGAG			
Caspase 1	Mouse	F: GATGGCATTAAGAAGGCCCA	60	229	NM_009807.2
		R: CCCTATCAGCAGTGGGCATC			
IL-1β	Mouse	F: GGGCCTCAAAGGAAAGAATCT	60	195	NM_008361.4
		R: GAGGTGCTGATGTACCAGTTGG			
α-SMA	Mouse	F: CCACGAAACCACCTATAACAGC	60	236	NM_007392.3
		R: GGAAGGTAGACAGCGAAGCC			
Collagen I	Mouse	F: CTGACTGGAAGAGCGGAGAG	60	116	NM_007742.4
		R: CGGCTGAGTAGGGAACACAC			
GAPDH	Mouse	F: TGAAGGGTGGAGCCAAAAG	60	227	NM_008084.3
		R: AGTCTTCTGGGTGGCAGTGAT			
NLRP3	Human	F: AAGACAGGAATGCCCGTCTG	60	162	NM_004895.4
		R: CCATCTTAATGGGACTCACGG			
α-SMA	Human	F: CTTGAGAAGAGTTACGAGTTGC	60	141	NM_001613.3
		R: GATGCTGTTGTAGGTGGTTTC			
Collagen I	Human	F: AAGACAGTGATTGAATACAAAACCAC	60	132	NM_000088.3
		R: GGGAGTTTACAGGAAGCAGACAG			
GAPDH	Human	F: CATCATCCCTGCCTCTACTGG	60	259	NM_002046.5
		R: GTGGGTGTCGCTGTTGAAGTC			

*Abbreviations*: *NLRP3* nod-like receptor protein-3, *Caspase 1* cysteinyl aspartate specific proteinase, *IL-1β* interleukin, *α-SMA* α-smooth muscle actin, *GAPDH* glyceraldehyde-3-phosphate dehydrogenase, *T* temperature

### Western blot and ELISA

Liver tissues and cells were lysed in RIPA Lysis buffer (Beyotime). Next, 40 μg of protein was loaded and boiled at 95 °C for 5 min, followed by fractionation using sodium dodecyl sulphate polyacrylamide gel electrophoresis (SDS-PAGE). The protein was transferred to PVDF membranes and then blocked with 5% non-fat milk in tris-buffered saline with tween 20 (TBST) before immune detection with the following primary antibodies: NLRP3 (1:500, Abcam, Cambridge, UK), Caspase 1 (1:500, Novus Biologicals, Littleton, CO, USA), IL-1β (1:500, Bioss, Beijing, China), α-SMA/ACTA2 (1:1000, Abcam), Collagen I (1:1000, Abcam), NF-κB (1:1000, CST, Danvers, MA, USA) and GAPDH (1:1000, CST). These antibodies were incubated overnight at 4 °C and HRP-conjugated secondary antibodies (1:5000, Aspen) were incubated for 1 h at room temperature. The protein was then labeled with enhanced chemiluminescent substrate (ECL, Millipore), and the band densities were detected by ImageJ software v.1.44 using the GAPDH band for standardization. All primary antibodies were rabbit anti-mouse IgG antibodies. All secondary antibodies were goat anti-rabbit IgG antibodies.

The serum was harvested and the IL-1β level was measured with a mouse IL-1β enzyme linked immunosorbent assay (ELISA) kit (eBioscience, San Diego, USA) according to kit instructions.

### Statistical analysis

Data were shown as the means ± standard error of mean (SEM). They were analyzed by SPSS 17.0 or GraphPad Prism 5.0 software. Student’s t-test was performed to determine differences between two groups. One-way ANOVA was used to evaluate all other differences among multiple groups. *P* < 0.05 indicated statistical significance.

## Results

### Pro-fibrotic markers depended on NLRP3 protein activation in LX-2 cells induced by SEA *in vitro*

**Fig. 1:**
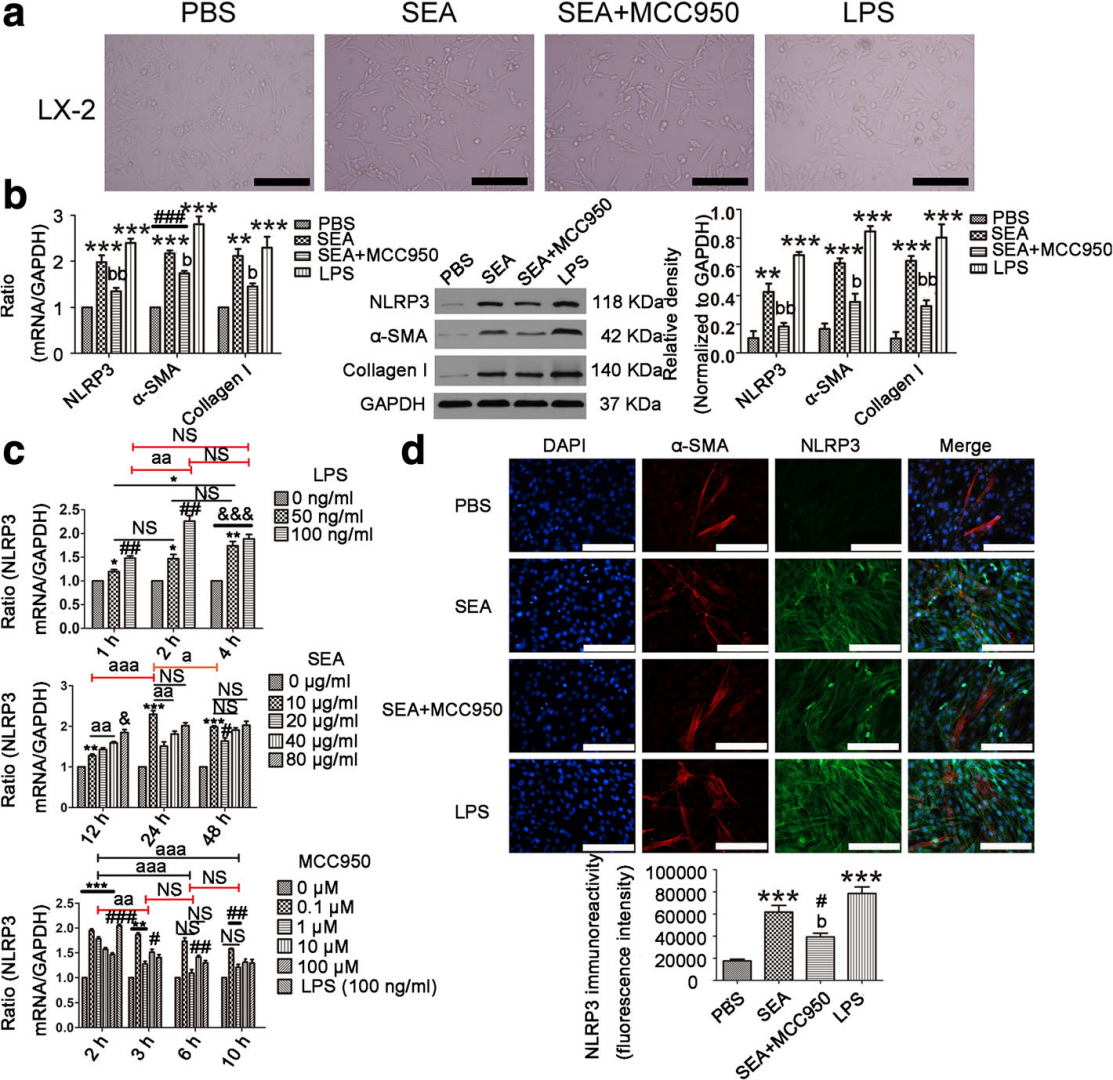
Pro-fibrotic markers and NLRP3 were both reduced by MCC950 in LX-2 cells triggered by SEA. **a** Morphology of LX-2 cells in different treatment groups observed under a microscope. **b** The mRNA and protein expressions of NLRP3, α-SMA and Collagen I were determined by RT-PCR and western blot, respectively. Relative quantitative western blot analysis of NLRP3, α-SMA and Collagen I was performed. Each immunoreactive band was digitized and normalized to loading control. **c** LX-2 cells were incubated with LPS (50 and 100 ng/ml) (top) or SEA (10, 20, 40, 80 μg/ml) (middle) for indicated time. LX-2 cells were pre-incubated with MCC950 (0.1, 1, 10, 100 μM) (bottom) for 0, 1, 4, 8 h, and then subjected to LPS (100 ng/ml) treatment for another 2 h. NLRP3 mRNA expression was determined by RT-PCR. ^*, #, a^*P* < 0.05, ^**, ##, aa^*P* < 0.01, ^***, ###, &&&, aaa^*P* < 0.001. **d** MCC950 (1 μM) pre-treatment for 4 h. This was then supplemented with SEA in LX-2 cells for an additional 24 h. LX-2 cells were treated with LPS for 2 h. Immunofluorescence staining of NLRP3 and α-SMA. Immunofluorescence staining of NLRP3 was digitized and normalized to control. Three independent experiments were performed, and one representative figure is shown (**b, d**). ^*, #^*P* < 0.05, ^**^P < 0.01, ^***, ###^*P* < 0.001 *vs* PBS; ^b^*P* < 0.01, ^bb^*P* < 0.01 *vs* SEA. *Abbreviation*: NS, no statistical significance. *Scale-bars*: **a**, 200 μm; **d**, 100 μm

Liver fibrosis was caused by schistosome eggs located in the portal system of the liver and was accompanied by inflammatory responses. SEA was released from schistosome eggs, which led to schistosomiasis-induced liver fibrosis (SSLF) [6]. LX-2 cells were used to study liver fibrosis because they are spontaneously immortalized in low serum conditions [42]. NLRP3 is a major proinflammatory “danger” receptor [43] and we wished to determine whether it was responsible for liver fibrosis. To directly address the role of NLRP3 during the development of SSLF, we first investigated whether SEA was sufficient to drive NLRP3 and pro-fibrotic markers (α-SMA and Collagen I) to increase *in vitro*. When we stimulated the LX-2 cells with SEA/LPS *in vitro* (Fig. 1a), we discovered that mRNA (NLRP3: *F*_(3,8)_ = 42.15, *P* < 0.0001; α-SMA: *F*_(3,8)_ = 59.83, *P* < 0.0001; Collagen I: *F*_(3,8)_ = 17.44, *P* = 0.0002) and protein (NLRP3: *F*_(3,8)_ = 39.38, α-SMA: *F*_(3,8)_ = 37.08, Collagen I: *F*_(3,8)_ = 26.61; all *P* < 0.0001) expressions of NLRP3, α-SMA and Collagen I were upregulated (Fig. 1b). Moreover, mRNA expression of NLRP3 exhibited a dose-dependent response in LX-2 cells that were treated with LPS after 1 h (*F*_(2,6)_ = 43.55, *P* = 0.0003), 2 h (*F*_(2,6)_ = 56.48, *P* = 0.0001) and 4 h (*F*_(2,6)_ = 39.53, *P* = 0.0004; Fig. 1c). The results suggest that NLRP3 protein and pro-fibrotic markers were upregulated in HSCs stimulated by SEA *in vitro*.

To verify that α-SMA and Collagen I production relied on the activation of NLRP3 inflammasome, we inhibited NLRP3 expression with MCC950. The mRNA (*F*_(3,8)_ = 42.15, *P* < 0.0001) and protein (*F*_(3,8)_ = 39.38, *P* < 0.0001) expressions of NLRP3 in the MCC950-treated cells were lower than those in the MCC950-untreated cells (Fig. 1b). As shown in Fig. 1d, similar results were obtained using immunofluorescence double staining. NLRP3 protein expression was upregulated in the LX-2 cells treated with SEA/LPS, and the SEA-treated group effect was reversed by MCC950 (*F*_(3,8)_ = 30.93, *P* < 0.0001; Fig. 1d). In addition, to determine the maximum expression of NLRP3 for the experiment, the optimal concentration and time of LPS, SEA and MCC950 was measured by RT-PCR. Experimental data indicated that the optimal concentration and time for LPS, SEA and MCC950 was 100 ng/ml and 2 h, 10 μg/ml and 24 h, and 1 μM and 4 h, respectively (Fig. 1c). In our experiments, LX-2 cells were treated with the above optimal concentration and time of LPS, SEA and MCC950. The results showed that the activation of NLRP3 led to an increase in α-SMA and Collagen I production, and this could be reduced by MCC950 in SEA-treated group (Fig. 1). This suggests that SEA induced α-SMA and Collagen I production is dependent on the expression of NLRP3 protein in LX-2 cells.

### ECM deposition depended on NLRP3 inflammasome activation in the liver of *S. japonicum-*infected mice *in vivo*

**Fig. 2:**
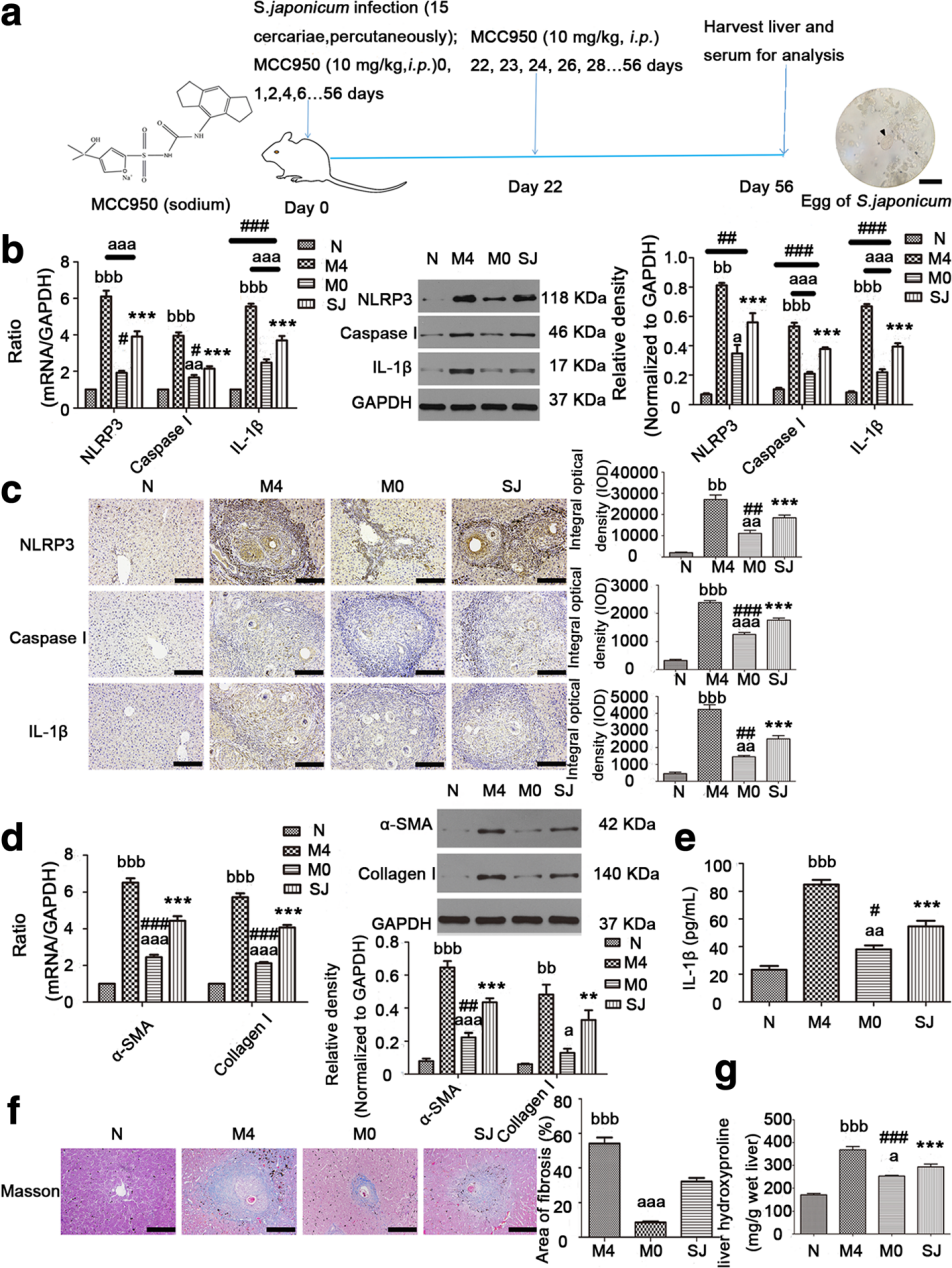
NLRP3 inflammasome expression and ECM deposition in the liver were both altered by MCC950 *in vivo*. BALB/c mice were infected percutaneously with 15 *S. japonicum* cercaria. In the M0 group, infected mice received i.p. injections of MCC950 (10 mg/kg) in 0.9% NaCl for 8 weeks starting on the day of infection. In the M4 group, infected mice received i.p. injections of MCC950 (10 mg/kg) in 0.9% NaCl for 5 weeks starting at day 22 post-infection. All the mice were sacrificed in order to harvest liver samples at 8 weeks after infection. **a** Chemical structure of MCC950 (sodium) (left). Experimental protocol of MCC950 application in liver fibrosis from infected mice (middle). Egg of *S. japonicum* (right). The mRNA and protein expressions of NLRP3, Caspase I, IL-1β (**b**), α-SMA and Collagen I (**d**) were determined by RT-PCR and western blot, respectively. The representative band images and the quantification were displayed. Liver tissues were fixed and stained with anti-NLRP3, Caspase I, IL-1β antibody (**c**) and stained with Masson trichrome (**f**), respectively. The quantitative changes were detected by computer-assisted morphometric analysis. **e** Levels of IL-1β in the plasma were detected by ELISA. **g** MCC950 affected on the contents of liver hydroxyproline. Data are mean ± SEM of 6 mice/group. ^#^*P* < 0.05, ^**, ##^*P* < 0.001, ^***, ###^*P* < 0.001, *vs* N; ^a^*P* < 0.05, ^aa, bb^*P* < 0.01, ^aaa, bbb^*P* < 0.001 *vs* SJ. *Abbreviations*: N, control group; SJ, infected group; NS, no statistical significance. *Scale-bars*: **a**, 50 μm; **c**, **f**, 100 μm

To further elucidate the role of NLRP3 inflammasome in the process of SSLF, we performed a mouse model using *S. japonicum*. BALB/c mice were infected with 15 cercariae through the abdominal skin for 56 days (Fig. 2a). The infected mice exhibited increased mRNA (NLRP3: *F*_(3,20)_ = 104.6, Caspase I: *F*_(3,20)_ = 91.18, IL-1β: *F*_(3,20)_ = 132.9, α-SMA: *F*_(3,20)_ = 167.4, Collagen I: *F*_(3,20)_ = 245.4; all *P* < 0.0001) and protein (NLRP3: *F*_(3,20)_ = 51.83, Caspase I: *F*_(3,20)_ = 143.9, IL-1β: *F*_(3,20)_ = 203.5, α-SMA: *F*_(3,20)_ = 81.15, Collagen I: *F*_(3,20)_ = 19.12; all *P* < 0.0001) expressions of NLRP3 inflammasome (Fig. 2b, c) and liver fibrosis markers (α-SMA and Collagen I) (Fig. 2d). In general, NLRP3 inflammasome was more broadly detected in close proximity to areas containing egg granulomas (Fig. 2c). IL-1β in serum could also be detected by ELISA (Fig. 2e). Collectively, these results suggest that NLRP3 inflammasome formation in the liver and liver fibrosis were the result of egg deposition and that the inflammasome contributed to the pathogenesis of schistosomiasis.

To further study the relationship between NLRP3 inflammasome and liver fibrosis during *S. japonicum* infection, we infected BALB/c mice in a similar way, but infected mice then received i.p. injections of MCC950 or vehicle, on the day of infection (M0 group) or at day 22 post-infection (M4 group) (experimental design summarized in Fig. 2a). Livers of the infected mice with SSLF exhibited substantial increases in NLRP3 inflammasome and ECM deposition (Fig. 2b-f). MCC950 injections in the M0 group reduced mRNA and protein expressions when compared with vehicle-treated infected mice (SJ group). However, MCC950 injections in the M4 group aggravated NLRP3 inflammasome and ECM deposition (Fig. 2b-f). As seen in Fig. 2f, the percentage of the fibrosis area showed a larger positive area for collagen staining in the liver of infected mice (SJ group) compared to control mice. This was smaller in the M0 group but larger in the M4 group (*F*_(3,20)_ = 91.12, *P* < 0.0001). Hyp content in the liver was indirectly measured for deposition of tissue collagen. These data showed Hyp content was increased in the liver of *S. japonicum-*infected mice, and this was counteracted by MCC950 in the M0 group but increased by MCC950 in the M4 group (*F*_(3,20)_ = 64.2, *P* < 0.0001; Fig. 2g). These results demonstrate that ECM deposition was synchronized with the expression of NLRP3 inflammasome. We speculate that ECM deposition depended on NLRP3 inflammasome expression.

### MCC950 altered conventional markers indicating pathological changes in mice infected with *S. japonicum*

**Fig. 3:**
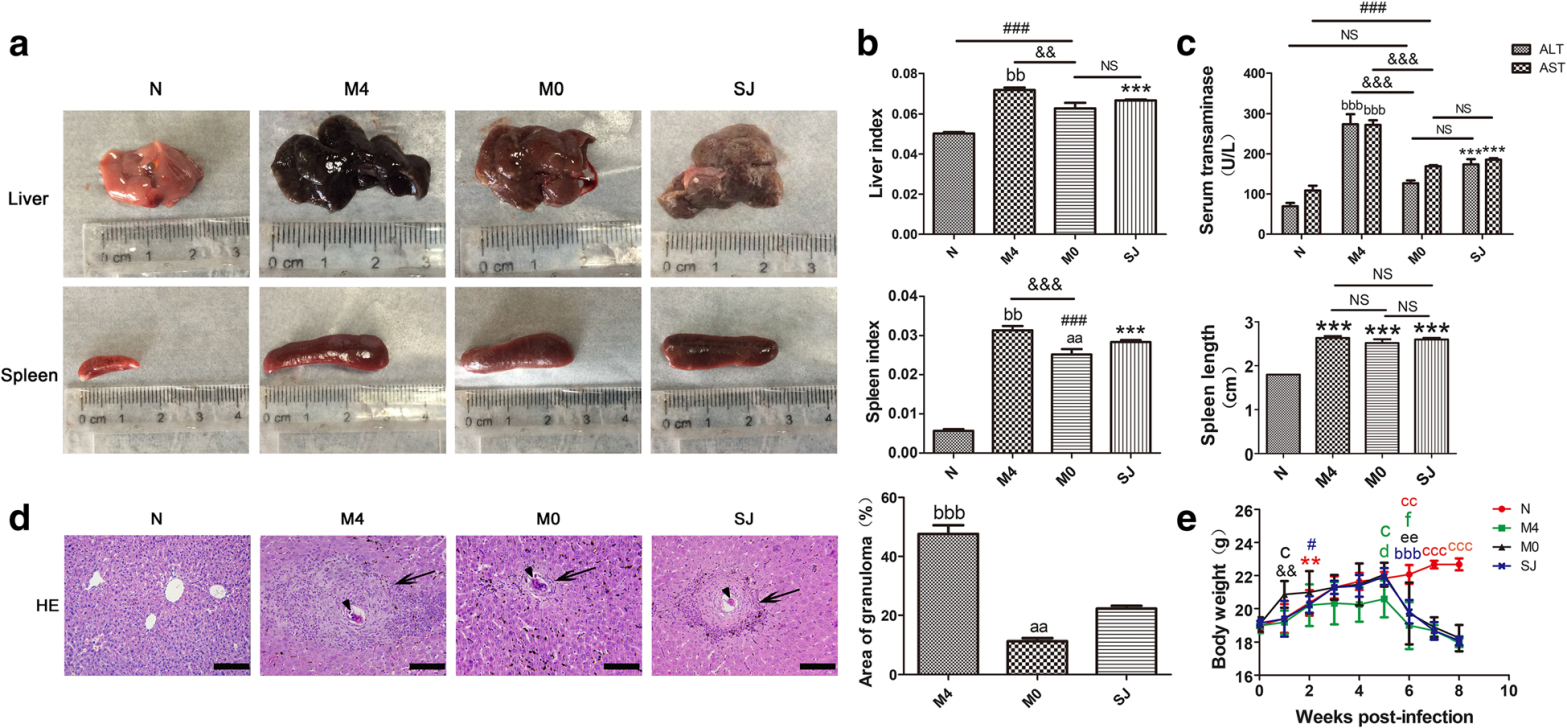
MCC950 affected the levels of ALT/AST, liver granuloma and body weight of infected mice. **a** Gross appearance of the liver and spleen from all mice. **b** Liver and spleen indexes, and spleen length. **c** MCC950 affected the serum transaminases (ALT/AST) levels of infected mice. **d** Liver tissues were fixed and stained with HE. Arrows indicate granulomatous lesions and arrowheads indicate schistosome eggs. The quantitative analyses were conducted under phase contrast microscope. **e** Dynamic curves of the body weights from different group mice. ^#, d^*P* < 0.05, ^**,&&^*P* < 0.01*vs* 0W; ^f^*P* < 0.05, ^ee^*P* < 0.01, ^bbb^*P* < 0.001 *vs* 5W; ^c^*P* < 0.05, ^cc^*P* < 0.01, ^ccc^*P* < 0.001. Data were mean ± SEM of 6 mice/group. (**b-d**) ^***,###^*P* < 0.001 *vs* N; ^aa^*P* < 0.01, ^bbb^*P* < 0.001 *vs* SJ; ^&&^*P* < 0.01, ^&&&^*P* < 0.001 *vs* M4. *Abbreviations*: N, control group; SJ, infected group; NS, no statistical significance. *Scale-bars*: **d**, 100 μm

The infected mice exhibited typical clinical symptoms, including anemia, hepatosplenomegaly, liver granuloma and fibrosis, and ascites [2]. To observe the conventional influences of MCC950 on liver disease caused by *S. japonicum*, all mice were first weighed and then sacrificed. The gross morphology of the liver and spleen, liver and spleen indexes and liver function were evaluated and analyzed. Normal livers displayed a bright red color, whereas the liver of the infected mice appeared a dark red and had many small white granulomatous nodules on the surface. Compared with the SJ group, the liver surface of the MCC950-treated (M0 group) mice had fewer white nodules, whereas livers from mice in the M4 group had more obvious white spots and the liver itself was a darker red (Fig. 3a). Liver and spleen indexes of SJ mice showed an increase over control mice (Fig. 3b). MCC950 treatment (M0 group) reversed this increase, whereas MCC950 treatment (M4 group) greatly increased the liver and spleen indexes over the SJ group (liver index: *F*_(3,20)_ = 33.22, spleen index: *F*_(3,20)_ = 156.6; both *P* < 0.0001). The spleen was enlarged at 56 days after infection, but there were no statistically significant differences among these three groups (SJ, M0 and M4 groups) (*P* > 0.05, Fig. 3b). Plasma ALT and AST increased substantially at 56 days in mice infected with *S. japonicum* compared to control mice (ALT: *F*_(3,20)_ = 32.47, AST: *F*_(3,20)_ = 59.97; both *P* < 0.0001; Fig. 3c). The liver function (ALT and AST) in serum was higher in the M4 group when compared to the SJ group. However, administration of MCC950 (M0 group) could not have abrogated the increase in ALT and AST on the day of infection in SSLF mice (Fig. 3c). Liver granulomas are a typical pathological hallmark of schistosomiasis. To evaluate the influences of MCC950 on *S. japonicum* egg-induced liver granuloma, HE-staining of liver sections was used. Control livers showed normal cellular organization of uninfected hepatic lobules, with the typical actinomorphous distribution of hepatic cords around central veins and little collagen around periportal areas (Fig. 3d). Infected livers showed a markedly altered histological structure. At 56 days post-infection, inflammatory granulomatous lesions were seen in the liver tissue around schistosome eggs, and the amount of collagen fibers were remarkably increased, with most being found around granulomas. MCC950 treatment markedly reduced the area of granulomas in the M0 group. Nevertheless, compared with SJ group, the area of liver granulomas from the M4 group was significantly larger (*F*_(3,20)_ = 98.74, *P* < 0.0001; Fig. 3d). In addition, body weight showed a sharp drop in infected mice (SJ, M0 and M4 groups) from the 35th to 42nd day, followed by more gradual weight loss, while control mice showed a steady gain in body weight until the 56th day (Fig. 3e). There were no statistically significant differences in body weight among SJ, M0 and M4 groups at the 56th day (*P* > 0.05), but a higher weight gain rate was observed at the 7th day in the M0 group (*F*_(3,20)_ = 4.678, *P* = 0.0124) and a lower weight was observed at the 35th day in the M4 group (*F*_(3,20)_ = 5.153, *P* = 0.0084) when compared to control mice (Fig. 3e). Thus, the liver surface of mice in the MCC950 treatment group (M0 group) was improved. Additionally, liver and spleen indexes, egg-induced liver granuloma and liver function were significantly reduced in the M0 group. In contrast, these indicators in the M4 group showed a more obvious increase than in the SJ group (Fig. 3).

### Egg burden and pairing of adult worms were similar in MCC950-treated and untreated mice infected with *S. japonicum*

**Fig. 4:**
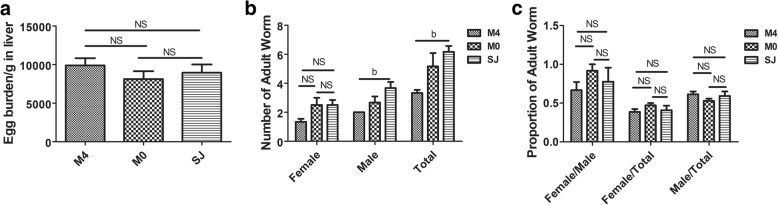
Egg burden, and proportion and percentage of female and male worms were similar. Egg burden (**a**), proportion and percentage of female and male worms (**c**) were similar in MCC950-treated and untreated mice infected with *S. japonicum*. **b** Numbers of female, male and total adult worms in mesenteric veins were analyzed in MCC950 treated and untreated infected mice. Data are mean ± SEM of 6 mice/group. ^b^*P* < 0.05 *vs* SJ. *Abbreviations*: SJ, infected group; NS, no statistical significance

The pathological differences in the liver of mice infected with *S. japonicum* depended on the time of MCC950 administration. The liver pathology of mice infected with *S. japonicum* was reduced by MCC950 administered on the day of infection, whereas it exacerbated the pathology when MCC950 was administered at day 22 post-infection. The SEA released by *S. japonicum* eggs resulted in a granulomatous formation, suggesting that egg load in the liver determines the severity of liver pathology. We evaluated the egg burden (Fig. 4a) and the number, proportion and percentage of female and male adult worms (Fig. 4b, c) in the mesenteric veins from MCC950-treated and untreated mice infected with *S. japonicum*. Results showed that the number of male and total *S. japonicum* was dramatically lower in the M4 group compared to infected mice that did not receive this treatment. Interestingly, liver egg burden (*F*_(3,20)_ = 0.7777, *P* = 0.4771), number of females (*F*_(3,20)_ = 0.7777, *P* = 0.4771), and proportion (*F*_(3,20)_ = 0.614, *P* = 0.5542) and percentage (female: *F*_(3,20)_ = 1.057, *P* = 0.372; male: *F*_(3,20)_ = 1.057, *P* = 0.372) of female and male *S. japonicum* were all similar among infected mice (Fig. 4). This result implies that MCC950 treatment does not affect the egg burden in the liver and the differential effect of MCC950 on hepatic injury was mainly caused by administration time.

### MCC950 altered pro-fibrotic markers in primary HSCs derived from *S. japonicum*-infected mice

**Fig. 5:**
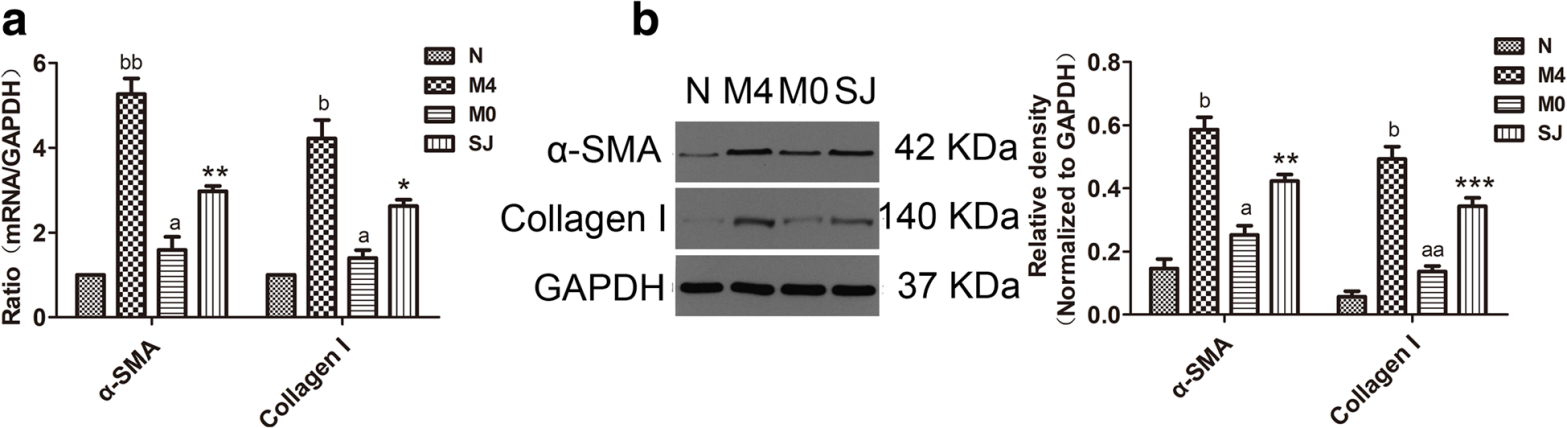
MCC950 altered pro-fibrotic markers in primary HSCs isolated from mice infected with *S. japonicum*. The primary HSCs were isolated from different group mice. The mRNA and protein expressions of α-SMA and Collagen I were determined by RT-PCR (**a**) and western blot (**b**), respectively. The representative images and quantification are shown. Three independent experiments were performed, and one representative figure is shown. ^*^P < 0.05, ^**^P < 0.01, ^***^P < 0.001 *vs* N; ^a, b^*P* < 0.05, ^aa, bb^*P* < 0.01 *vs* SJ

The pro-fibrotic markers play a major role in liver fibrosis of schistosomiasis [3, 6]. To further explore whether MCC950 affected pro-fibrotic markers, we separated primary HSCs from the livers of *S. japonicum*-infected mice. As illustrated in Fig. 5, the expressions of pro-fibrotic markers’ (α-SMA and Collagen I) mRNA (α-SMA: *F*_(3,8)_ = 58.15, Collagen I: *F*_(3,8)_ = 34.06; both *P* < 0.0001) and protein (α-SMA: *F*_(3,8)_ = 40.62, Collagen I: *F*_(3,8)_ = 55.27; both *P* < 0.0001) were dramatically increased in the SJ group when compared to the control group. This was reduced by MCC950 in the M0 group but increased in the M4 group when compared to the SJ group using RT-PCR (Fig. 5a) and western blotting (Fig. 5b).

### NLRP3 inflammasome expression in KCs was more involved in liver fibrosis than in HSCs in mice infected with *S. japonicum*

**Fig. 6:**
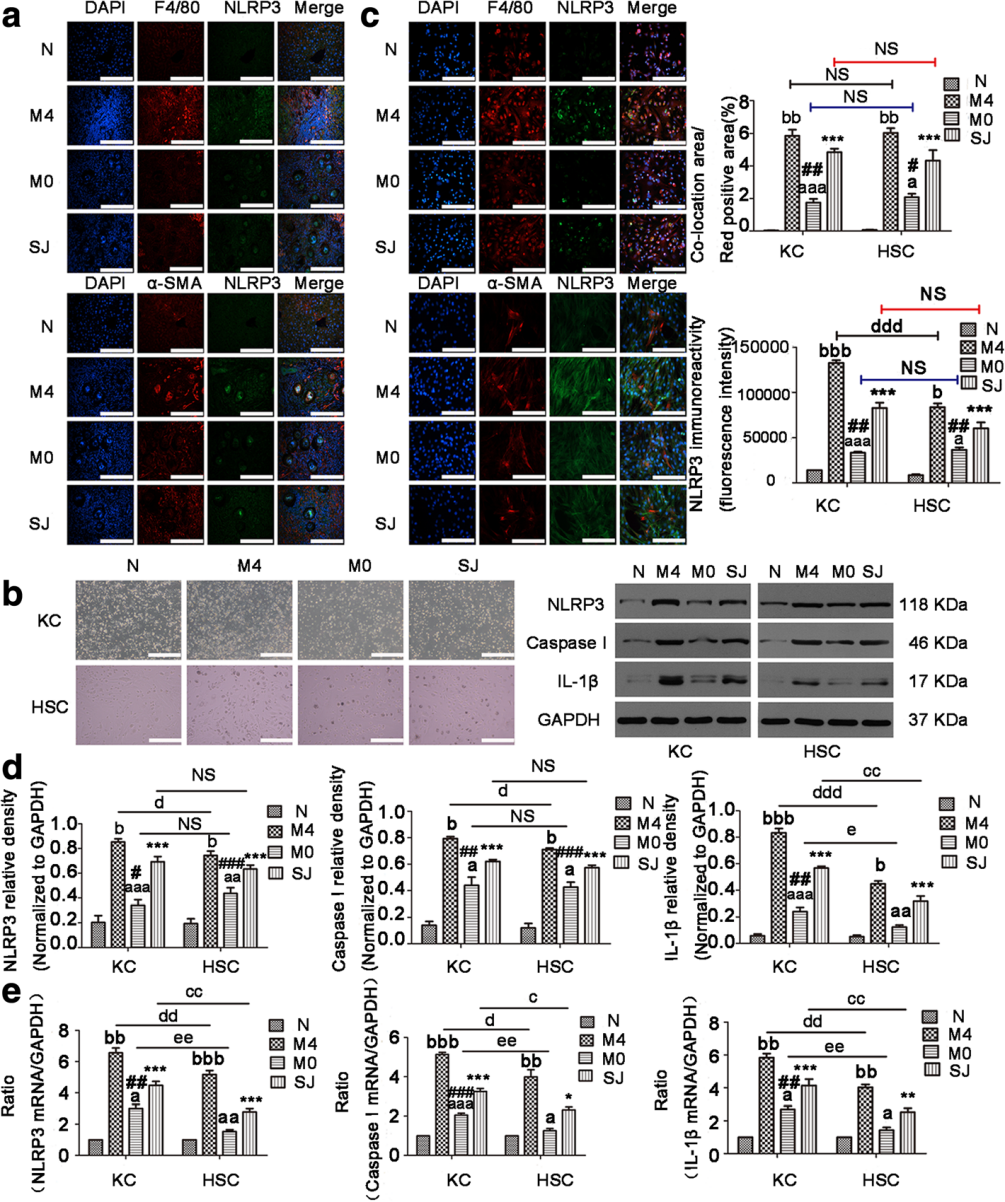
NLRP3 inflammasome expression in KCs was more involved in liver fibrosis than in HSCs. NLRP3 immunofluorescent co-staining with F4/80 (KCs marker) (top), or with α-SMA (HSCs marker) (bottom) in liver tissues (**a**) and in primary KCs or HSCs (**c**) from mice. The quantitative changes were analyzed by Image-Pro Plus 6.0. **b** The primary KCs and HSCs were isolated from all mice. Morphology of primary KCs and HSCs was observed under a microscope. The mRNA and protein expressions of NLRP3, Caspase I, and IL-1β in primary KCs and HSCs were detected by RT-PCR (**e**) and western blot (**d**), respectively. The expressions of NLRP3, Caspase I, and IL-1β protein were quantified. Data are mean ± SEM of 6 mice/group. ^*, #^*P* < 0.05, ^**, ##^*P* < 0.01, ^***, ###^*P* < 0.001 *vs* N; ^a, b^*P* < 0.05, ^aa, bb^*P* < 0.01, ^aaa, bbb^*P* < 0.001 *vs* SJ. ^c, d, e^*P* < 0.05, ^cc, dd, ee^*P* < 0.01, ^ddd^*P* < 0.001. *Abbreviations*: N, control group; SJ, infected group; NS, no statistical significance. *Scale-bars*: **a**, 100 μm; **b**, 200 μm; **c**, 100 μm

In our experiments we observed that MCC950 can reduce NLRP3 expression in both LX-2 cells and primary HSCs. NLRP3 inflammasome was widely expressed in KCs and HSCs. These two kinds of cells both play an essential role in liver fibrosis. To better demonstrate which kind of cells originated from NLRP3 inflammasome implicated in hepatic fibrosis (particularly in KCs or HSCs in mice infected with *S. japonicum*), we examined the co-localization of NLRP3 with the KCs marker F4/80 or with the HSCs marker α-SMA in liver tissues. As shown in Fig. 6a, the co-localization area percentage of NLRP3 with F4/80 (*F*_(3,20)_ = 118.8, *P* < 0.0001), or with α-SMA (*F*_(3,20)_ = 49.9, *P* < 0.0001) in target cells was significantly increased surrounding liver granulomas in the infected mice when compared to control mice. Additionally, these measures were decreased in the M0 group but enhanced in the M4 group. However, there was no statistically significant difference in the expression of NLRP3 protein between primary KCs and HSCs within the same group from the SJ (*t*_(4)_ = 0.7622, *P* = 0.4884), M0 (*t*_(4)_ = 1.038, *P* = 0.3578) and M4 (*t*_(4)_ = 0.3865, *P* = 0.7188) groups.

To further survey the effects of NLRP3 inflammasome expression in KCs and HSCs, cells were successfully separated from the livers at the indicated time (Fig. 6b). NLRP3 with F4/80 in KCs or with α-SMA in HSCs were labelled by immunofluorescence double staining (Fig. 6c), and NLRP3, Caspase 1 and IL-1β were all detected in KCs and HSCs by RT-PCR and western blotting (Fig. 6d, e). The above results showed an enhancement in the mRNA and protein levels of NLRP3, Caspase1 and IL-1β in primary KCs (NLRP3 mRNA: *F*_(3,8)_ = 92.16, NLRP3 protein: *F*_(3,8)_ = 146, Caspase1 mRNA: *F*_(3,8)_ = 292.1, Caspase1 protein: *F*_(3,8)_ = 62.16, IL-1β mRNA: *F*_(3,8)_ = 67.27, IL-1β protein: *F*_(3,8)_ = 192.8; all *P* < 0.0001) and HSCs (NLRP3 mRNA: *F*_(3,8)_ = 108.8, NLRP3 protein: *F*_(3,8)_ = 118, Caspase1 mRNA: *F*_(3,8)_ = 44.15, Caspase1 protein: *F*_(3,8)_ = 83.8, IL-1β mRNA: *F*_(3,8)_ = 63.65, IL-1β protein: *F*_(3,8)_ = 55.3; all *P* < 0.0001) from the SJ group in comparison to control mice. NLRP3 inflammasome was downregulated in the M0 group but was markedly upregulated in the M4 group when compared to the SJ group. The mRNA of NLRP3 inflammasome expression was higher in KCs than in HSCs for SJ (NLRP3: *t*_(4)_ = 4.787, *P* = 0.0087; Caspase1: *t*_(4)_ = 4.229, *P* = 0.0134; IL-1β: *t*_(4)_ = 3.487, *P* = 0.0252), M0 (NLRP3: *t*_(4)_ = 5.181, *P* = 0.0066; Caspase1: *t*_(4)_ = 5.427, *P* = 0.0056; IL-1β: *t*_(4)_ = 4.776, *P* = 0.0088) and M4 (NLRP3: *t*_(4)_ = 4.787, *P* = 0.0087; Caspase1: *t*_(4)_ = 3.053, *P* = 0.0379; IL-1β: *t*_(4)_ = 6.218, *P* = 0.0034) groups (Fig. 6e). The NLRP3 inflammasome protein expression was also higher in KCs than in HSCs for the M4 group (NLRP3: *t*_(4)_ = 4.410, *P* = 0.0116; Caspase1: *t*_(4)_ = 4.490, *P* = 0.0109; IL-1β: *t*_(4)_ = 10.02, *P* = 0.0006) (Fig. 6c, d). The protein expression of IL-1β was higher in KCs than in HSCs for the SJ (*t*_(4)_ = 5.967, *P* = 0.004) and M0 (*t*_(4)_ = 3.307, *P* = 0.0297) groups, as well as for the M4 (*t*_(4)_ = 10.02, *P* = 0.0006) group (Fig. 6d). Taken together, the present study raises the possibility that NLRP3 inflammasome is activated and formed in both primary KCs and HSCs in *S. japonicum-*infected mice. Nonetheless, KCs as immune cells were more sensitive to NLRP3 inflammasome expression than HSCs.

### NF-κB was involved in NLRP3 inflammasome-induced liver fibrosis in mice infected with *S. japonicum*

**Fig. 7:**
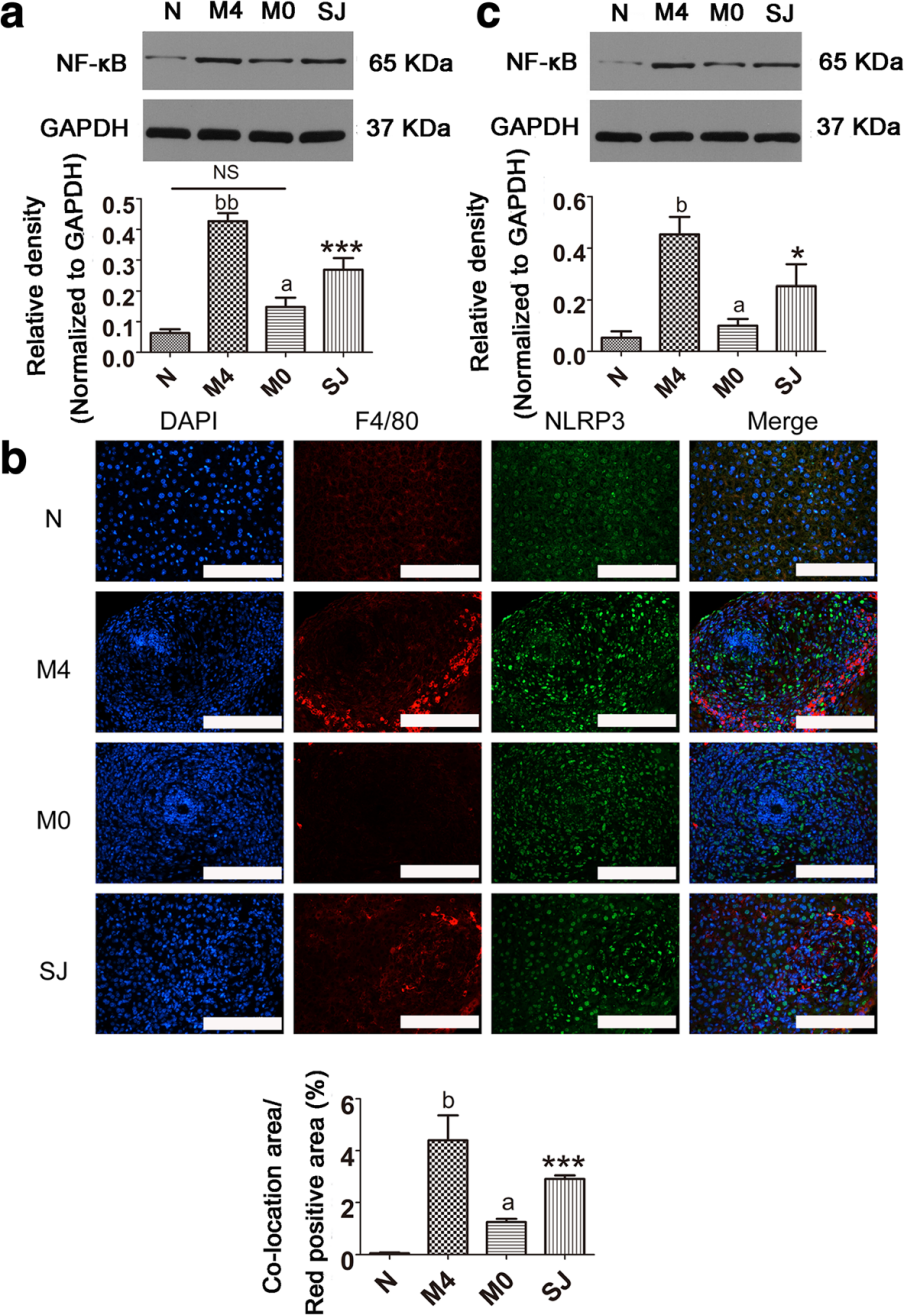
NF-κB was involved in NLRP3 inflammasome-induced liver fibrosis of mice infected with *S. japonicum*. Protein expression of NF-κB in liver tissues (**a**) and in primary KCs (**c**) isolated from model mice were detected *via* western blot. The expression of NF-κB protein was quantified. **b** NF-κB immunofluorescent co-staining with F4/80 (KCs marker) in liver tissues. The quantitative changes were measured by Image-Pro Plus 6.0. Data are mean ± SEM of 6 mice/group. ^a^*P* < 0.05, ^b^*P* < 0.05, ^bb^*P* < 0.01 *vs* SJ; ^*^P < 0.05, ^***^P < 0.001 *vs* N. *Abbreviations*: N, control group; SJ, infected group; NS, no statistical significance. *Scale-bars*: **b**, 100 μm

NF-κB is primarily composed of p50 and p65 subunits. NF-κB is part of a key signal transduction pathway and is generally used to study infection, inflammation and fibrosis through p65 subunit nuclear transfer [44]. To further elucidate the mechanism by which infection with *S. japonicum* induces NLRP3-dependent liver fibrosis, we examined the role of NF-κB in this process. We attempted to detect the NF-κB p65 subunit protein in the liver tissues from all mice. As expected, the NF-κB p65 protein was significantly increased in the SJ group compared to control mice. By contrast, the NF-κB p65 protein showed a decreased presence in the M0 group but strong expression in the M4 group relative to the SJ group (*F*_(3,20)_ = 30.92, *P* < 0.0001; Fig. 7a). As NLRP3 inflammasome participation in liver fibrosis triggered by *S. japonicum* is principally derived from KCs, we analyzed the co-localization of NF-κB and the KCs marker F4/80 in liver tissues. The expression of NF-κB protein in KCs was significantly increased in mice infected with *S. japonicum*. Interestingly, the NF-κB p65 protein in KCs was decreased when MCC950 was applied on the day of infection, but was displayed an opposite trend when MCC950 was given at day 22 post-infection (*F*_(3,20)_ = 45.62, *P* < 0.0001; Fig. 7b). To further understand the expression of NF-κB protein in KCs, the NF-κB p65 protein was examined in primary KCs by western blotting. We found that the expression of NF-κB p65 protein in primary KCs was similar to that found in liver tissues (*F*_(3,20)_ = 30.04, *P* = 0.0001; Fig. 7c). Collectively, NF-κB p65 expression is responsible for the effects of MCC950 on liver fibrosis. We speculate that NF-κB might be downstream of NLRP3 inflammasome, which then participates in NLRP3 inflammasome-induced liver fibrosis in mice infected with *S. japonicum*.

## Discussion

Chronic inflammation and inflammasome activation are crucial factors in the pathogenesis of liver fibrosis [16, 18]. To date, the NLRP3 inflammasome is reported to be one of the most-studied inflammasomes with respect to its role in antivirus responses [45], antifungal responses [36] and various human diseases [46, 47]. From the present study, it is reasonable to conclude that ECM deposition depended on NLRP3 inflammasome expression in mice infected with *S. japonicum via* NF-κB. We therefore propose that there are four main issues to be addressed. The first issue involves whether NLRP3 inflammasome can be activated in *S. japonicum-*infected mice and expressed in LX-2 cells evoked by SEA from *S. japonicum* eggs. The second problem relates to how MCC950 can alter NLRP3 inflammasome and liver fibrosis of *S. japonicum* differently depending on the time of MCC950 administration. The third issue deals with how the NLRP3 inflammasome is principally involved in liver fibrosis of *S. japonicum* derived from KCs. Finally, we tentatively put forward that NF-κB is a key point for NLRP3 inflammasome induced ECM deposition.

We confirm that NLRP3 inflammasome was expressed in liver fibrosis of mice infected with *S. japonicum*. NLRP3 inflammasome expression was investigated in liver tissues and in primary KCs and HSCs isolated from *S. japonicum*-infected mice. Our results largely support those of Tang and colleagues [17, 22]; however, NLRP3 protein was only detected in the LX-2 cells induced by SEA. Nevertheless, SEA alone was unable to induce other components of the NLRP3 inflammasome in the LX-2 cells. Similar data was gathered in bone marrow-derived DCs (BMDCs) treated with SEA alone [48]. However, contradictory results have been reported that NLRP3 inflammasome is expressed in LX-2 cells induced by LPS [49], mouse primary HSCs induced by SEA from *S. japonicum* eggs [17], and BMDCs induced by SEA from *S. mansoni* eggs following TLR-stimulation [48]. NLRP3 inflammasome expression is usually regulated by activated TLR [28]. LPS, acting as a ligand for TLR4, could induce a small amount of NLRP3 inflammasome expression [32]. We speculate that SEA is a multi-component complex with different effects on different cells. LX-2 cells derived from humans may be less vulnerable to SEA induced NLRP3 inflammasome activation.

The results reported here indicate that NLRP3 inflammasome activation plays an essential function in SSLF. NLRP3 inflammasome and ECM deposition were decreased by MCC950 in the M0 group, whereas they were increased by MCC950 in the M4 group *in vivo*. Parallel experiments showed NLRP3 inflammasome and pro-fibrotic markers had a weak expression in LX-2 cells co-treated with SEA and MCC950, as well as in primary KCs and HSCs from the M0 group. However, these markers showed a strong expression in the above-mentioned primary cells from the M4 group. Using MCC950 and NLRP3 siRNA, NLRP3 inflammasome and liver fibrosis can be reduced in nonalcoholic steatohepatitis (NASH) and *S. japonicum* mice, respectively [22, 50]. Using MCC950, similar findings were also obtained in other disease model including diabetic encephalopathy, hemorrhagic brain damage and lupus nephritis [51–53]. NLRP3 protein was not affected while Caspase I and IL-1β were both reduced when MCC950 was used in LPS and ATP co-induced mouse bone marrow derived macrophages (BMDM) [38]. Currently, MCC950 has dual effects on the NLRP3. One view is that MCC950 only affects NLRP3 inflammsome activation. The other view is that MCC950 not only affects NLRP3 inflammsome activation, but also affects the NLRP3. This difference may be related to different treatment methods. NLRP3 was reduced by MCC950 in our experiments, which may be attributed to blocking the upstream signal of NLRP3, like NEK7 [54, 55]. At present, MCC950 is used in the Ang-II induced cardiac fibrosis mouse model, and a reduction in fibrosis is also observed [56]. Several researchers pointed out that NLRP3 inflammsome deficiency has a protective effect against carbon tetrachloride (CCl_4_) or thioacetamide (TAA)-induced liver fibrosis, and that it reduces mortality and liver injury after acetaminophen administration [57, 58]. The development of liver fibrosis induced by CCl_4_ and TAA was apparently delayed in mice lacking NLRP3 or ASC, and the upregulation of Collagen I and TGF-β induced by MSU could not be observed in ASC^-/-^ HSCs [57]. Likewise, it has been reported that NLRP3 inflammasome activation was required for fibrosis development in NLRP3 knockout and mutant mice [18, 59]. Similar results were found in liver fibrosis after bile duct ligation (BDL) or Ang-II infusion [21]. We believe that hepatic fibrosis induced by *S. japonicum* is due to the presence of the eggs [2]. To understand whether NLRP3 inflammasome activation induced ECM deposition in a *S. japonicum* mouse model, we sought to block NLRP3 expression. We thought it was better to block NLRP3 inflammasome at two time points. One time point was at the beginning of *S. japonicum* infection and the second was prior to *S. japonicum* egg production. We found that ECM deposition was differently affected depending on the time of MCC950 administration. ECM deposition was alleviated when MCC950 was administered at the beginning of infection but was augmented when MCC950 was administered before *S. japonicum* egg production. We propose that the decrease in ECM deposition at the first time point was due to MCC950 inhibiting inflammation. The results were similar to other disease models, including NASH [50], BDL and Ang-II [21]. Our results are again consistent with the results obtained by Tang and colleagues, who used NLRP3 siRNA technology in the same mouse model [22]. The expression of kidney fibrosis markers, MCC950 administration on the seventh day, was similar to the non-administered group in adenine-enriched diet-induced kidney fibrosis [60]. This result is possibly due to inflammation-driven fibrosis. We hypothesize that MCC950 administration led to an increase in ECM deposition at the later time point and that this was too late to block inflammation and hence promoted liver fibrosis. Liver inflammation and fibrosis have an interacting relationship in that inflammation is the initiator of liver fibrosis, and liver fibrosis is the ultimate outcome of excessive inflammation [12, 61]. When *S. japonicum*-infected mice were administered MCC950 before egg production, it was the equivalent to suppressing the excessive inflammatory response in the liver. Undoubtedly, liver fibrosis in the M4 group should be exacerbated. The results in the present study suggest that during the period of *S. japonicum* infection, inflammasome activation is accompanied with fibrogenic changes in the liver.

Our results show that NLRP3 inflammasome in KCs and HSCs are both implicated in the progression of liver fibrosis in mice infected with *S. japonicum*. Interestingly, NLRP3 inflammasome derived from KCs is larger than in HSCs. Using immunofluorescence double staining, we identified that the co-localization area percentage of NLRP3 with F4/80, or with α-SMA in target cells was increased in liver tissues and in primary KCs and HSCs from the SJ group in comparison to control mice. This could be suppressed by MCC950 in the M0 group but reinforced by MCC950 in the M4 group. We confirmed this in primary KCs and HSCs *via* RT-PCR and western blot. Additionally, there is substantial evidence that NLRP3 inflammasome activation and formation is present in KCs and HSCs. By comparing the expression of the NLRP3 inflammasome in the above two kinds of cells, we found that the NLRP3 inflammasome expression in KCs was generally higher than in HSCs. KCs and HSCs are pivotal for chronic inflammation and fibrogenesis [4, 12, 37, 61]. The effect of macrophages and HSCs on liver fibrosis depends on their activation state [37, 62]. The NLRP3 inflammasome has been reported to amplify chronic liver inflammation and activate HSCs [18]. NLRP3 inflammasome was expressed not only in HSCs [21, 57] but also in KCs [50, 63]. *In vitro* and *in vivo* experiments showed cholesterol crystals [50] or LPS [32] could induce KCs to release IL-1β, and this process can be inhibited by MCC950 [50]. Currently, toxicological profiling of metal oxide nanoparticles (TMO) has a similar effect in KCs [63]. These results support the view that activation of NLRP3 inflammasome contributes to liver fibrosis of *S. japonicum* infected mice in both KCs and HSCs. Our study showed that NLRP3 inflammasome activation mostly comes from KCs. We recognize that there are some limitations in our study, in that we did not examine other mouse strains, the activity and numbers of *S. japonicum* cercariae, and other time points of *S. japonicum* infection.

We have demonstrated that NF-κB is involved in NLRP3 inflammasome induced liver fibrosis of *S. japonicum-*infected mice. Our results indicate that NF-κB expression is increased in liver tissues and in KCs of the SJ group compared to the control group, and this was suppressed by MCC950 in the M0 group but enhanced by MCC950 in the M4 group. NF-κB, as a transcription factor, regulates liver inflammation and takes part in the development of liver fibrosis [37]. The traditional view is that NF-κB is upstream to the NLRP3 inflammasome [28, 30], but in our study we found that NF-κB was regulated by NLRP3 inflammasome. The expression of NF-κB was blocked by NLRP3 inhibitor MCC950 and NLRP3 gene knockout, in the model of inflammatory hyperalgesia and chronic unpredictable mild stress (CUMS) induced depression, respectively [64, 65]. It was also reduced following NLRP3 knocked down in the early stage of *Staphylococcus aureus* infection by siRNA in human monocytic cell line THP-1 [66]. The latest papers report that attenuating the degradation of NF-κB inhibitor (IκB) could decrease collagen synthesis following silencing of NLRP3 in lung fibrosis [67]. These studies demonstrate that NF-κB and NLRP3 inflammasome have an upstream and downstream relationship with each other.

**Fig. 8:**
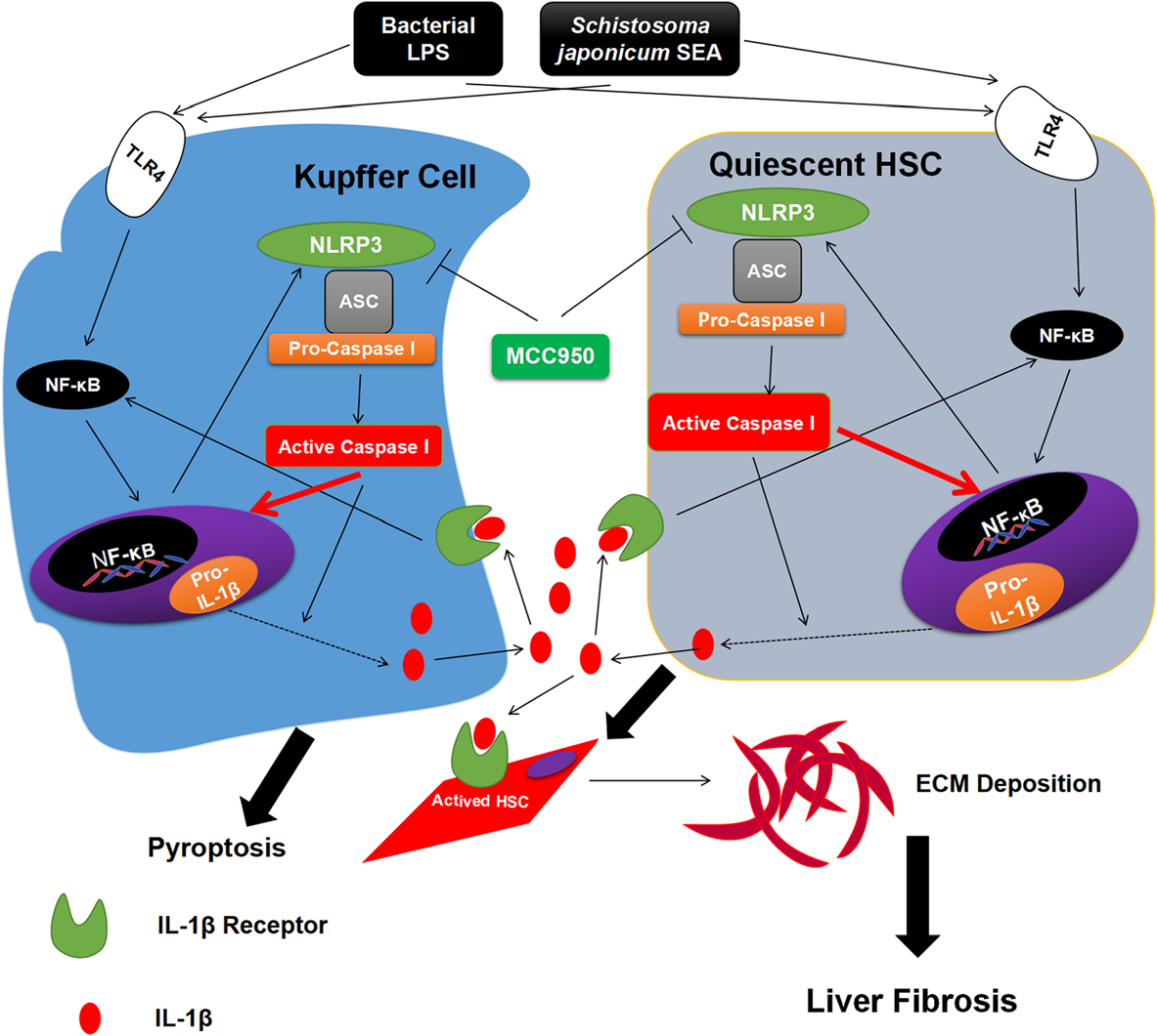
Proposed mechanism of *S. japonicum* SEA-mediated NLRP3 inflammasome activation involved in KCs and HSCs activation. As pathogen-associated molecular patterns (PAMPs), *S. japonicum* SEA is involved in the inflammatory crosstalk between KCs and HSCs, and TLR4-dependent plays a crucial role in liver inflammation during the progression of liver fibrosis. The present study applied LPS/SEA to show the involvement of the NLRP3 inflammasome pathway in the secretion of IL-1β both from KCs and HSCs. IL-1β generated by inflammasome activation will bind to receptors located on the above two kinds of cells, and upregulate fibrotic markers, resulting in their activation and ECM deposition, and leading to liver fibrosis. MCC950 could directly bind to NLRP3 and block NLRP3, suggesting that MCC950 might represent a potential preventing target for liver fibrosis

In addition, liver function (ALT/AST) and liver granuloma induced by the eggs were both altered by MCC950, but different time points of administration led to different results. AST recovery level was not as good as ALT in the M0 group, which may result in statistical differences between the N and M0 groups. ALT is mainly distributed in the liver and is expressed in the cytoplasm of hepatocytes, while AST is mainly distributed in the myocardium, followed by the liver, and is expressed in the mitochondria [68]. Therefore, AST is not as sensitive to ALT when the liver is affected. ALT/AST was reduced by MCC950 administration for 56 days in methionine/choline deficiency (MCD) diet induced non-alcoholic fatty liver disease (NAFLD) mice [50]. Our previous administration results are similar to these results. MCC950 could also reduce the number of worms. Male worms are more numerous than female worms, but single males are more susceptible than females to MCC950, which are less likely to survive in MCC950 administration. The numbers of male worms were reduced more obviously owing to significant sex differentiation in the M4 group, but the mechanism needs to be further elucidated. To our knowledge, we are the first to use MCC950 in the liver fibrosis mouse model caused by *S. japonicum*. We tentatively propose that the relationship between MCC950 and liver fibrosis is triggered by *S. japonicum* eggs (Fig. 8).

So far, our conclusion is that the NLRP3 inflammasome mainly participates in the inflammatory response in the acute phase of *S. japonicum* infection and induces immune cells KCs to produce a large number of cytokines such as IL-1β [50], IL-6 [50], IL-17 [7], IL-18 [69] and TGF-β [69]. These massively released cytokines alter the immune microenvironment of the liver and evoke cascading signal responses through the cytokine receptors itself on the surface of the cell membrane [70]. These induce the resting hepatic stellate cells to activate and to convert into myofibroblasts, eventually forming ECM deposition and leading to liver fibrosis [4, 6]. Moreover, there is no evidence that NLRP3 inflammasome is directly involved in the process of liver fibrosis.

## Conclusions

In summary, we have demonstrated that the NLRP3 inflammasome takes part in the process of *S. japonicum* induced liver fibrosis *via* NF-κB signaling. The results indicate that the activation of NLRP3 inflammasome is both in KCs and HSCs, with a more robust effect in KCs. We surmise that the activation of NLRP3 inflammasome in KCs may be followed by the production of cytokines, for instance IL-1β. This then activates HSCs accompanied by NLRP3 inflammasome activation, and then results in ECM deposition and liver fibrosis. The data provide new arguments about the development of liver fibrosis following *S. japonicum* infection. We have also identified novel molecular targets that could have the potential to be developed into preventative measures for SSLF. In the future, we may further explore whether there are other signaling pathways involved in NLRP3 inflammasome induced liver fibrosis following *S. japonicum* infection.

## Abbreviations

ALT: Alamine aminotransferase
Ang-II: Angiotensin II
ASC: Apoptosis-associated speck-like protein containing a caspase recruitment domain
ASH: Alcoholic steatohepatitis
AST: Aspartate aminotransferase
BDL: Bile duct ligation
BMDCs: Bone marrow-derived DCs
BMDM: Mouse bone marrow derived macrophages
BSA: Bovine serum albumin
CARD: Caspase recruitment domain-containing protein 8
Caspase 1: Cysteinyl aspartate specific proteinase
CCl4: Carbon tetrachloride
CRID3: Cytokine release inhibitory drug 3
CUMS: Chronic unpredictable mild stress
Cy3: Cyanine 3.18
DAB: Streptavidin-peroxidase complex 3, 3-diaminobenzidine tetrahydroxychloride
DAMPs: Danger-associated molecular patterns
DMEM: Dulbecco’s modified Eagle’s medium
ECL: Enhanced chemiluminescent substrate
ECM: Extracellular matrix
ELISA: Enzyme linked immunosorbent assay
EPG: Eggs per gram
F4/80: Mouse epidermal growth factor-like module-containing mucin-like hormone receptor-like 1
FBS: Fetal bovine serum
FITC: Fluorescein isothiocyanate
GAPDH: Glyceraldehyde-3-phosphate dehydrogenase
H_2_O_2_: Hydrogen peroxide
HE: Hematoxylin-eosin
HSCs: Hepatic stellate cells
Hyp: Hydroxyproline
IL: Interleukin
IOD: Integrated optical density
IκB: Inhibitor of NF-κB
KCs: Kupffer cells
LPS: Lipopolysaccharide
MCD: Methionine/choline deficiency
NAFLD: Non-alcoholic fatty liver disease
NASH: Nonalcoholic steatohepatitis
NF-κB: Nuclear factor-κB
NLRP3: Nod-like receptor protein-3
NLRs: Nucleotide-binding oligomerization domain (NOD) leucine-rich repeat containing receptors
PAMPs: Pathogen-associated molecular patterns
PBS: Phosphate-buffered saline
PFA: Paraformaldehyde
PRRs: Pattern recognition receptors
RT-PCR: Real-time PCR
SDS-PAGE: Dodecyl sulphate polyacrylamide gel electrophoresis
SEA: Soluble egg antigen
SEM: Standard error of mean
SPF: Specific pathogen-free
SSLF: Schistosomiasis-induced liver fibrosis
TAA: Thioacetamide
TBST: Tris-buffered saline with tween 20
TGF-β: Transforming growth factor-β
TMO: Toxicological profiling of metal oxide nanoparticles
TNF-α: Tumor necrosis factor-α
α-SMA: α-smooth muscle actin
